# Efficacy and safety of Chinese herbal medicines combined with cyclophosphamide for connective tissue disease-associated interstitial lung disease: A meta-analysis of randomized controlled trials

**DOI:** 10.3389/fphar.2023.1064578

**Published:** 2023-02-23

**Authors:** Xietian Yin, Shichao Zhao, Nan Xiang, Jidong Chen, Jun Xu, Yudan Zhang

**Affiliations:** ^1^ College of the First Clinical, Hubei University of Chinese Medicine, Wuhan, China; ^2^ Department of Rheumatism Immunology, Hubei Provincial Hospital of TCM, Wuhan, China; ^3^ Department of Geriatrics, Hubei Provincial Hospital of TCM, Wuhan, China; ^4^ Hubei Provincial Key Laboratory of Occurrence and Intervention of Rheumatic Diseases, Hubei Minzu University, Enshi, China

**Keywords:** connective tissue disease-associated interstitial lung disease, metaanalysis, randomized controlled trials, cyclophosphamide, Chinese herbal medicine (CHM)

## Abstract

**Objectives:** To evaluate the effectiveness and safety of Chinese herbal medicines (CHMs) combined with cyclophosphamide (CTX) for connective tissue disease-associated interstitial lung disease (CTD-ILD) by performing a meta-analysis.

**Methods:** We searched RCTs of Chinese herbal medicines therapy for connective tissue disease-associated interstitial lung disease in ten databases. Methodological quality assessment was performed by the Cochrane collaboration tool. RevMan v5.3 and Stata v14.0 software were used for performing data analysis. This study was conducted and reported following the PRISMA checklist.

**Results:** Overall, seven RCTs with 506 participants were included for this analysis. Current data indicated that Chinese herbal medicines combined with cyclophosphamide contributed to a betterment in improving the clinical efficacy rate of connective tissue disease-associated interstitial lung disease [risk ratio (RR) = 1.21, 95% confidence interval (CI): (1.09, 1.35), *p* = 0.0003], tended to benefit improvement of lung function, which included VC [weighted mean difference (WMD) = 9.49, 95% CI: (5.54, 13.45), *p* < 0.00001], FVC [standardized mean difference (SMD) = 0.83, 95% CI: (0.36, 1.29), *p* = 0.0005], FEV1 [SMD = 0.54, 95% CI: (0.23, 0.86), *p* = 0.0008], TLC [SMD = 0.90, 95% CI: (0.68, 1.13), *p* < 0.00001], DLCO [SMD = 1.05, 95% CI: (0.38, 1.73), *p* = 0.002], and MVV [SMD = 0.83, 95% CI: (0.50, 1.17), *p* < 0.00001], and it also could significantly reduce the HRCT integral of lungs [SMD = −2.02, 95% CI: (−3.14, −0.91), *p* = 0.0004] and the level of ESR [WMD = −13.33, 95% CI: (−18.58, −8.09), *p* < 0.00001]. Furthermore, there was no statistical significance in the incidence of adverse events (AEs), which indicate that Chinese herbal medicines combined with cyclophosphamide is safe and does not increase adverse events compared with cyclophosphamide alone.

**Conclusion:** Our analysis indicates that Chinese herbal medicines combined with cyclophosphamide may be a more effective strategy on the treatment of connective tissue disease-associated interstitial lung disease in the clinic. Because it included studies with relatively small sample size, the results need to be confirmed by more well-designed and large-scale RCTs.

**Systematic Review Registration:** https://10.37766/inplasy2022.12.0010.

## Introduction

Connective tissue disease (CTD) generally refers to a group of autoimmune diseases involving the damage of blood vessels and connective tissue (Barnes et al., 2018). As the lung has the functions of metabolism and immune regulation and is rich in connective tissues such as blood vessels and collagen, it often becomes the target organ of CTD. Interstitial lung disease (ILD) is commonly seen in CTD patients with prevalence varying is 10%–30%, which is one of the serious causes of death in patients with CTD (Zhong et al., 2020). Connective tissue disease-associated interstitial lung disease (CTD-ILD) is more common in patients with systemic sclerosis (SSc), dermatomyositis/polymyositis (DM/PM), primary Sjogren’s syndrome (pSS), rheumatoid arthritis (RA) and systemic lupus erythematosus (SLE) (Lee and Lee, 2015). Due to the different primary diseases, CTD-ILD may show different clinical manifestations and pathological features, which makes its early diagnosis and treatment difficult. Clinically, multidisciplinary collaboration is often required for disease diagnosis and evaluation. Although many CTD-ILD patients may stay steady or progress slowly, some of those may develop progressive pulmonary fibrosis, which is seriously affecting the quality of patient life and lifetime. Therefore, in order to prevent adverse outcomes, it is imperative to carry out early accurate diagnosis and select appropriate individualized treatment for patients with CTD-ILD (Li Y. H. et al., 2021). At present, although the etiology and pathogenesis of CTD-ILD are not fully understood, it is clear that immune mediated chronic inflammation and lung injury in the disease early stage play a role in its development (Xing et al., 2021). Glucocorticoid (GC) and immunosuppressive therapy are the primary treatments for CTD-ILD patients in clinic. Immunosuppressive drugs usually include azathioprine, tacrolimus, mycophenolate, cyclophosphamide (CTX) and so on (Jee and Corte, 2019). At the same time, the use of pirfenidone, nidanib and biology agent are effective in preventing pulmonary fibrosis (Hatemi et al., 2018; Maher et al., 2020; Rahimi, 2020). However, the occurrence of adverse events (AEs) and the extremely high cost of these treatments can prevent prolonged follow up with the treatment in patients with CTD-ILD. Therefore, faced with the limitations of current treatment, a safer, cheaper and more effective therapeutic strategy need to be explored.

Nowadays, Chinese herbal medicines (CHMs) have drawn more and more attention from scholars because of its high efficacy and low incidence of AEs. CHMs are widely used in the treatment of CTD-ILD (Chen et al., 2010; Qin et al., 2018; Sun et al., 2018). In China, CTD-ILD belongs to the category of “Bi syndrome of Lung” or “Wei syndrome of Lung,” which we called “Fei Bi” or “Fei Wei.” According to Traditional Chinese medicine (TCM) theory, lung is closely related to kidney and blood vessel. TCM holds that lung advocate gas, faces the hundred vessels, kidney receive qi, meanwhile lung can regulate the circulation of qi and blood. Based on the relationship between lung, kidney and blood vessels, Tonifying lung and kidney, promoting blood circulation and removing blood stasis (known as Bufei yishen, Huoxue huayu) is the classic TCM therapy for CTD-ILD (Li and Qu, 2019). For example, Feng and Wang (Feng and Wang, 2019) found that when combined with Yangyin Yiqi Mixture and GC, it could improve the clinical treatment effect and improve the symptoms of patients. Xie (2011) also found that Bufei Tongluo Pill combined with methotrexate in the treatment of early CTD-ILD is effective in decreasing the area of interstitial lung lesions with a good safety. Some clinical studies have evaluated the therapeutic effect of CHMs on CTD-ILD and its positive effect on improving the lung function and optimizing the radiographic indicators in recent decades (Han and Zhang, 2014; Liu, 2017; Wang et al., 2019). However, there are no reliable evidence-based medical studies to confirm its efficacy for CTD-ILD, leading to its clinical benefits are still controversial. Therefore, to further confirm the clinical value of CHMs therapy for CTD-ILD, we conducted this meta-analysis to evaluate the efficacy and safety of combining CHMs with CTX in the treatment of CTD-ILD. Thus, it will provide a basis for clinical medication.

## Materials and methods

This study was a secondary study of the literature, ethical approval was not required, it was performed and reported according to the Preferred Reporting Items for Systematic Reviews and Meta-Analyses (PRISMA) guidelines (Page et al., 2021) and the completed checklist was presented in Supplementary Table S1. The protocol used in this study was registered in Inplasy on 3 December 2022 (Registration No: INPLASY2022120010).

### Data source and search strategy

Two trained investigators (by authors SCZ and JX) independently searched randomized controlled trials (RCTs) of CHMs therapy for CTD-ILD from the following ten different databases: PubMed, EMBASE, Web of science, Cochrane Library, ClinicalTrials.gov, China National Knowledge Infrastructure, Wanfang Database, China Biological Medicine Database, VIP Journals Database and Chinese Clinical Trial Register, which retrieved from their inception to 28 August 2022. On the other hand, additional relevant records were identified from published reviews and the reference lists of selected RCTs to avoid missing qualified studies. The search terms include: [(“Connective tissue disease”) OR (“Systemic sclerosis”) OR (“Dermatomyositis”) OR (“Sjogren’s syndrome”) OR (“Rheumatoid arthritis”) OR (“Systemic lupus erythematosus”)] AND [(“Interstitial lung disease”) OR (“Interstitial Pneumonia”)] AND [(“Chinese herbal drugs”) OR (“Chinese traditional medicine”) OR (“Chinese and western medicine”)]. For the Chinese databases, these strategies were translated into Chinese. If necessary, we would contact the original researcher to obtain more complete research data. The full search strategy in Embase was presented in Supplementary Table S2.

### Inclusion criteria

Studies were included if they fulfilled the following criteria: 1) Population: Patients were individuals who were diagnosed with CTD-ILD. We included only individuals who were definitive CTD as defined by recognized diagnostic criteria at the time of study determination. We included the CTDs commonly associated with ILD: SSc, DM/PM, pSS, RA and SLE; 2) Intervention and comparison: Experimental groups were combination treatment of CHMs and CTX, while control groups were treatment of CTX alone. GCs were used as background therapy in both experimental and control groups; 3) Outcome: primary outcomes were lung function [including vital capacity (VC), forced vital capacity (FVC), forced expiratory volume in one second (FEV1), total lung capacity (TLC), carbon monoxide diffusing capacity (DLCO), maximal voluntary ventilation per minute (MVV)], HRCT integral of lungs, secondary endpoints included clinical effective rate, erythrocyte sedimentation rate (ESR), number of adverse events (AEs); 4) Design: RCT, results available in either Chinese or English.

### Exclusion criteria

Articles were excluded if they fulfilled the exclusive criteria: 1) Study subjects did not meet CTD-ILD diagnostic criteria; 2) Both experimental and control groups contained CHMs only therapy, or the control group was treated with other CHMs, or inappropriate interventions; 3) Duplicated articles or animal experiments or reviews or unavailable data studies or irrelevance to outcome indicators; 4) Case reports or conference abstracts or comments or academic dissertations; 5) Non-RCT.

### Quality assessment and data extraction

Two independent reviewers (SCZ and JX) searched and screened the articles, meanwhile extracted the relevant data according to predefined criteria. The collected data included identification information (i.e., the first author, publishing year, country, and sample size), participant baseline characteristics (i.e., age, sex, and disease duration), details of experimental and control groups (i.e., intervention, comparison, and treatment duration), and outcome parameters. Two reviewers (SCZ and JX) individually estimated the methodological quality of the included trials based on the bias risk assessment tool of the Cochrane collaboration (Cumpston et al., 2019). The tool had 6 domains, each domain was ranked as “Low,” “Unclear” and “High.” The 6 domains were 1) Method of random allocation; 2) Allocation concealment; 3) Blinding method; 4) Integrity of data; 5) Selective reporting; 6) Other biases. If disagreements on the assessment were identified, the third author (NX) was asked for the final decisions.

### Data analysis

RevMan v5.3 and Stata v14.0 were used for this meta-analysis. For continuous data, standardized mean difference (SMD), weighted mean difference (WMD), and a 95% confidence internal (CI) were calculated. For dichotomous data, risk ratio (RR) and a 95% CI were calculated. Statistical heterogeneity was evaluated using the I^2^ and χ^2^ tests. When heterogeneity was identified (*I* ^*2*^ ≥ 50%), a random-effects model was selected, otherwise a fixed-effects model was applied. The source of the heterogeneity was identified by subgroup analysis and sensitivity analysis if heterogeneity exists. When *p* < 0.05, statistically significant differences were considered.

## Results

### Search results and study characteristics

**FIGURE 1:**
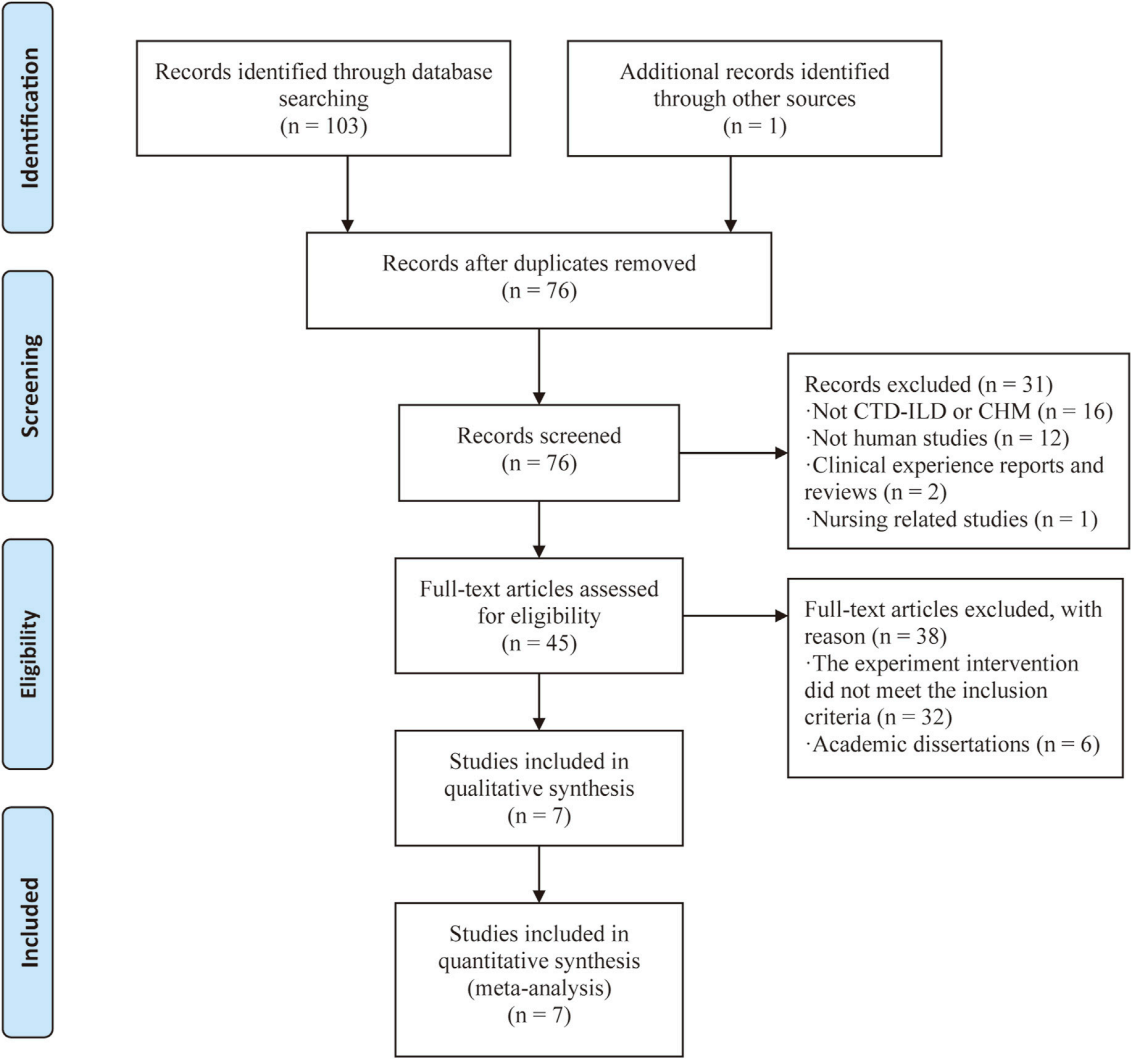
Study selection flow chart.

As Figure 1 shown, we found 104 relevant records reporting on CTD-ILD treatment using CHMs in our initial search. 28 records were discarded because of duplications. A total of 31 records were excluded due to inaccurate observation object, including 16 non CTD-ILD or CHMs studies, 12 non-human studies, 2 reviews, and 1 nursing study. After going through the full-text, another 32 records were discarded because of they did not meet the inclusion criteria, and 6 records were discarded because of academic dissertations, which may not be peer-reviewed. Finally, seven RCTs (Li et al., 2011; Bai et al., 2017; Wang et al., 2017; Huang et al., 2018; Zhao et al., 2018; Liu et al., 2020; Pan et al., 2020) with 506 participants (260 in the experimental group received CHMs plus CTX therapy and 246 in the control group received CTX treatment) were considered eligible for our meta-analysis. Both groups received GCs as background therapy. Among them, 2 studies were CTD-ILD, 4 were RA-ILD, and 1 were pSS-ILD. The general characteristics of these eligible studies were summarized in Table 1.

**TABLE 1:** The characteristics of the included studies.

Study Id	Sample size(T/C)	Age (years)		Gender (M/F)		Disease duration (years)		Intervention		Duration (months)	Outcome
		T	C	T	C	T	C	T	C		
Bai et al. (2017)	26/25	52.64 ± 6.89	54.91 ± 7.83	7/19	8/17	4.55 ± 2.06	3.98 ± 1.87	CHMs + CTX	CTX	3	a, b, d, f, g
Huang et al. (2018)	40/40	58.60 ± 7.69	58.48 ± 7.63	16/24	18/22	9.75 ± 1.42	9.82 ± 1.46	CHMs + CTX	CTX	6	a, d, e, f, h, i
Li et al. (2011)	23/22	56.34 ± 5.71	55.64 ± 8.92	5/18	4/18	6 ∼ 30	6 ∼ 29	CHMs + CTX	CTX	3	a, b, c, e, f, h, i, j
Liu et al. (2020)	30/30	46.7 ± 8.9	47.1 ± 7.5	12/18	10/20	8.5 ± 3.5	9.3 ± 4.1	CHMs + CTX	CTX	6	a, b, d, e, f, h, i, j
Pan et al. (2020)	26/22	60.46 ± 10.54	60.50 ± 10.41	11/15	9/13	4.73 ± 1.58	3.86 ± 1.48	CHMs + CTX	CTX	6	a, d, e, g
Wang et al. (2017)	60/60	57.1 ± 8.9	56.8 ± 9.4	18/42	20/40	9.3 ± 2.5	9.1 ± 2.2	CHMs + CTX	CTX	6	d, e, f, g, h, i
Zhao et al. (2018)	57/52	54.8 ± 7.9	53.6 ± 8.6	27/30	25/27	4.6 ± 1.7	4.5 ± 2.1	CHMs + CTX	CTX	3	a, c, d, e, h

T, treatment group; C, control group; M/F, male/female; CHMs, Chinese herbal medicines; CTX, cyclophosphamide; outcomes: a, clinical efficacy; b, AEs; c, VC; d, FVC; e, FEV1; f, TLC; g, DLCO; h, MVV; i, HRCT integral; j, ESR.

### Risk of bias

**FIGURE 2:**
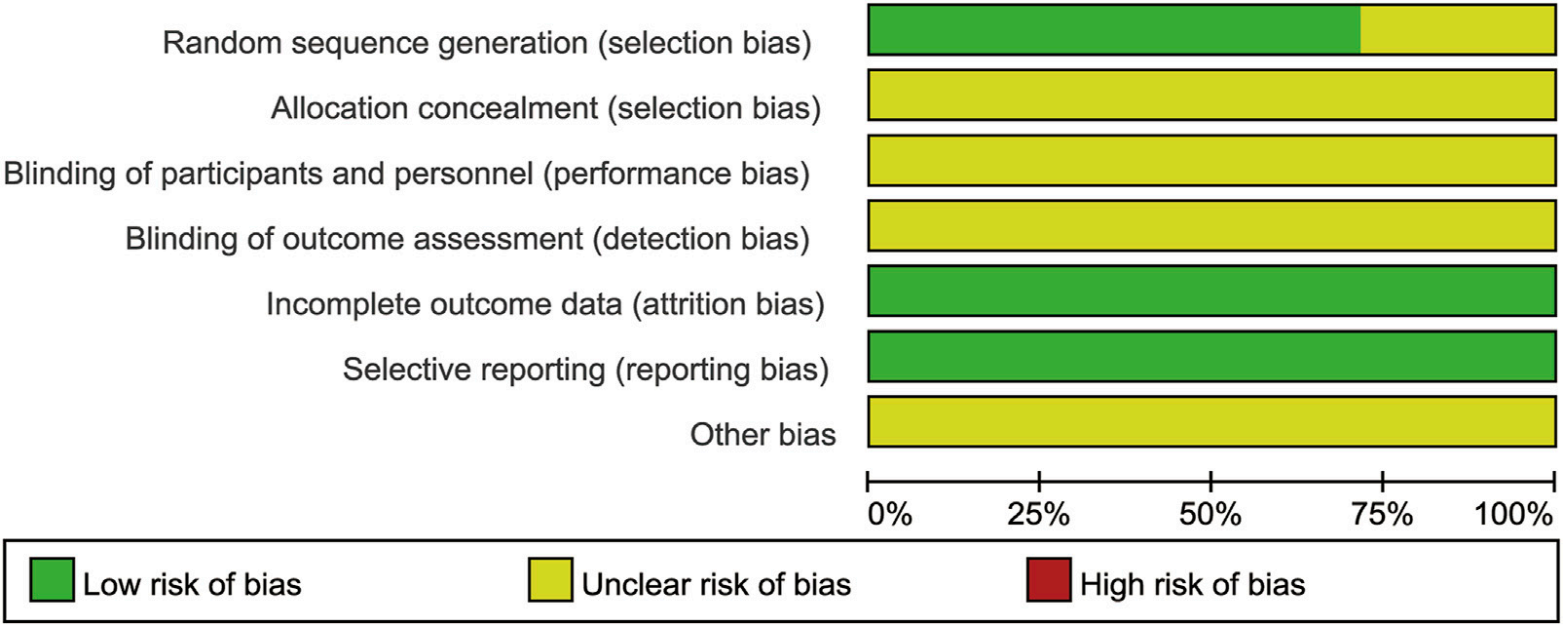
Bias risk graph.

**FIGURE 3:**
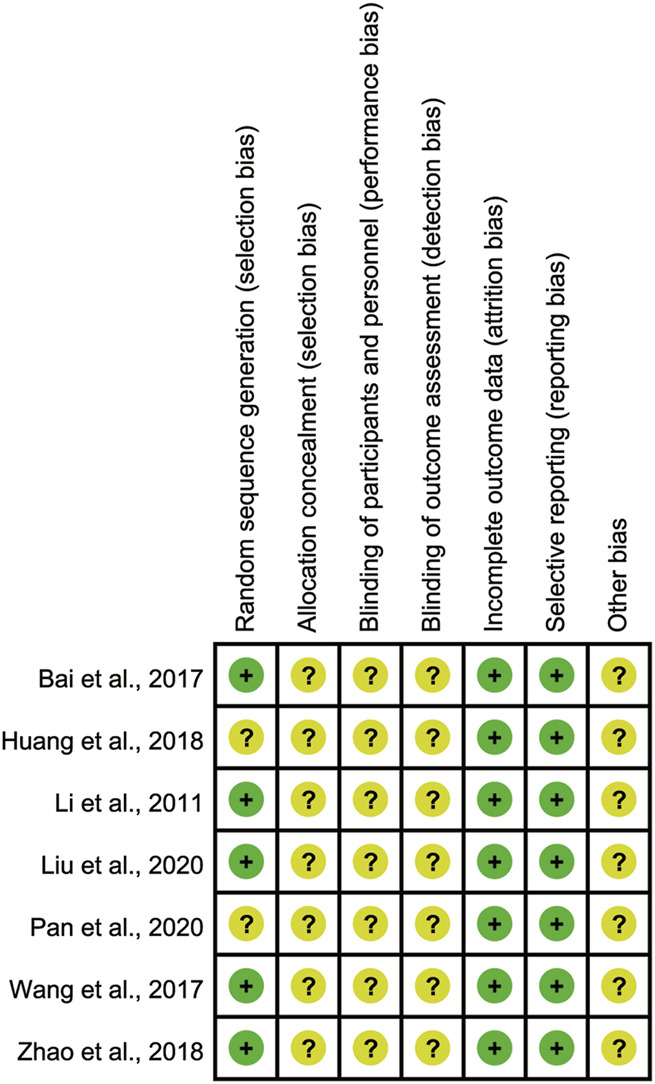
Bias risk summary.

As Figures 2, 3 shown, the potential sources of bias and the included articles methodological quality were outlined. All included trials claimed randomized, however, only 5 reported the randomization method (Li et al., 2011; Bai et al., 2017; Wang et al., 2017; Zhao et al., 2018; Liu et al., 2020). No study reported allocation concealment and blinding method. All studies had the complete outcome data, none of them had selective reporting. Other biases were not determined.

### Effects of intervention

#### Primary outcomes

##### Changes in lung function

VC, FVC, FEV1, TLC, DLCO, and MVV were measured to evaluate the improvement of lung function in CTD-ILD patients.

**FIGURE 4:**
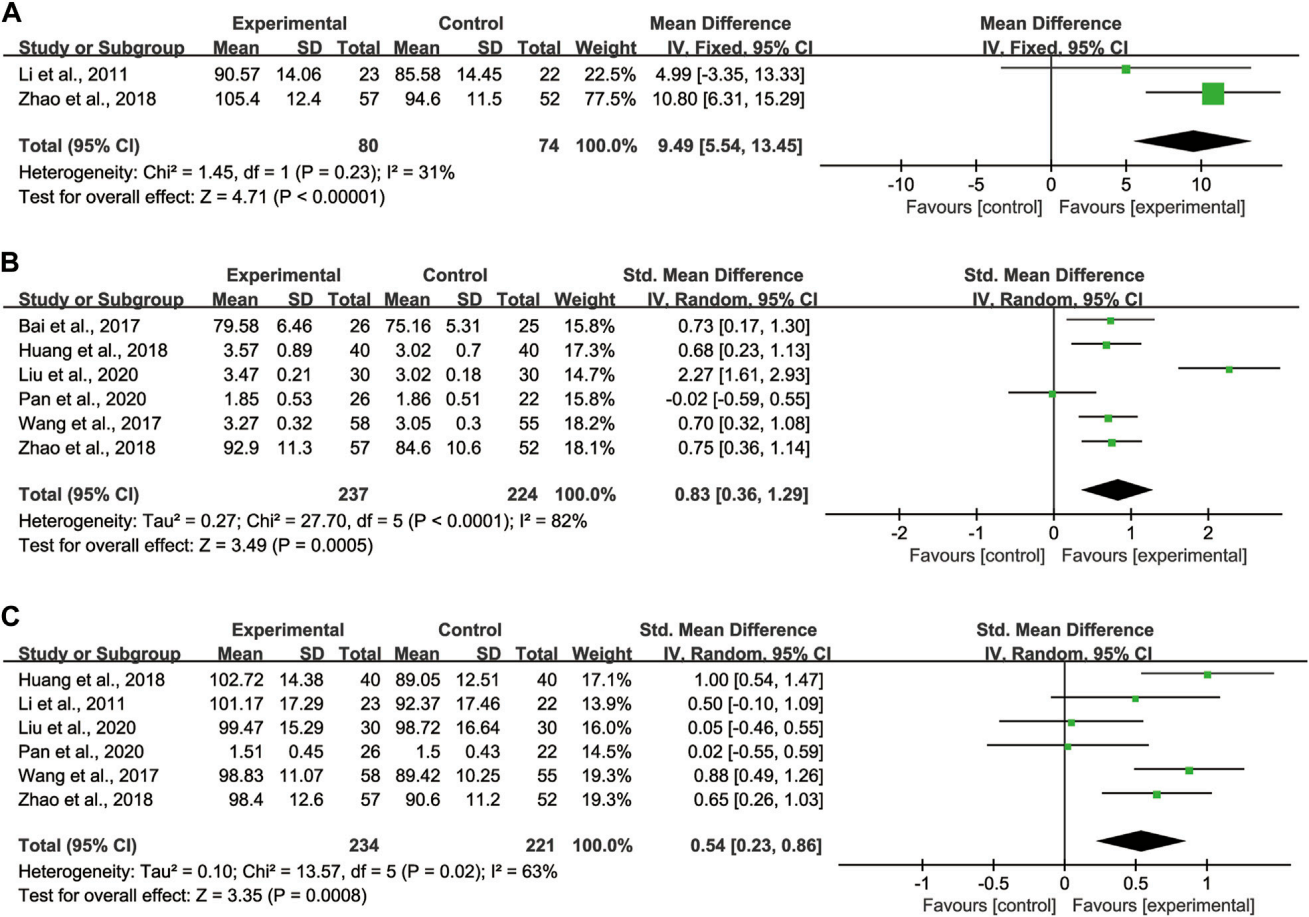
Forest plot of lung function: CHMs plus CTX treatment *versus* CTX treatment. **(A)** VC, **(B)** FVC, **(C)** FEV1.

Regarding VC, 2 studies (Li et al., 2011; Zhao et al., 2018) with totaling 154 patients (80 in the experimental group, 74 in the control group) were reported, as these studies showed no significant heterogeneity (*I* ^*2*^ = 31%), we adopted the fixed effect model (Figure 4A). Pooled analysis indicated that CHMs combined with CTX treatment had advantages in improving VC compared with CTX treatment [WMD = 9.49, 95% CI: (5.54, 13.45), *p* < 0.00001].

Regarding FVC, 6 studies (Bai et al., 2017; Wang et al., 2017; Huang et al., 2018; Zhao et al., 2018; Liu et al., 2020; Pan et al., 2020) with totaling 461 patients (237 in the experimental group, 224 in the control group) were reported, as data heterogeneity test was high (*I* ^*2*^ = 82%), we adopted the random effect model (Figure 4B). Pooled analysis indicated that CHMs combined with CTX treatment could significantly improve the FVC of patients [SMD = 0.83, 95% CI: (0.36, 1.29), *p* = 0.0005].

Regarding FEV1, 6 studies (Li et al., 2011; Wang et al., 2017; Huang et al., 2018; Zhao et al., 2018; Liu et al., 2020; Pan et al., 2020) with totaling 455 patients (234 in the experimental group, 221 in the control group) were reported, as we noted significant heterogeneity in these studies (*I* ^*2*^ = 63%), the random effect model was adopted (Figure 4C). According to the pooled analysis results in FEV1, we concluded that the CHMs combined with CTX group could improve the FEV1 of patients [SMD = 0.54, 95% CI: (0.23, 0.86), *p* = 0.0008].

**FIGURE 5:**
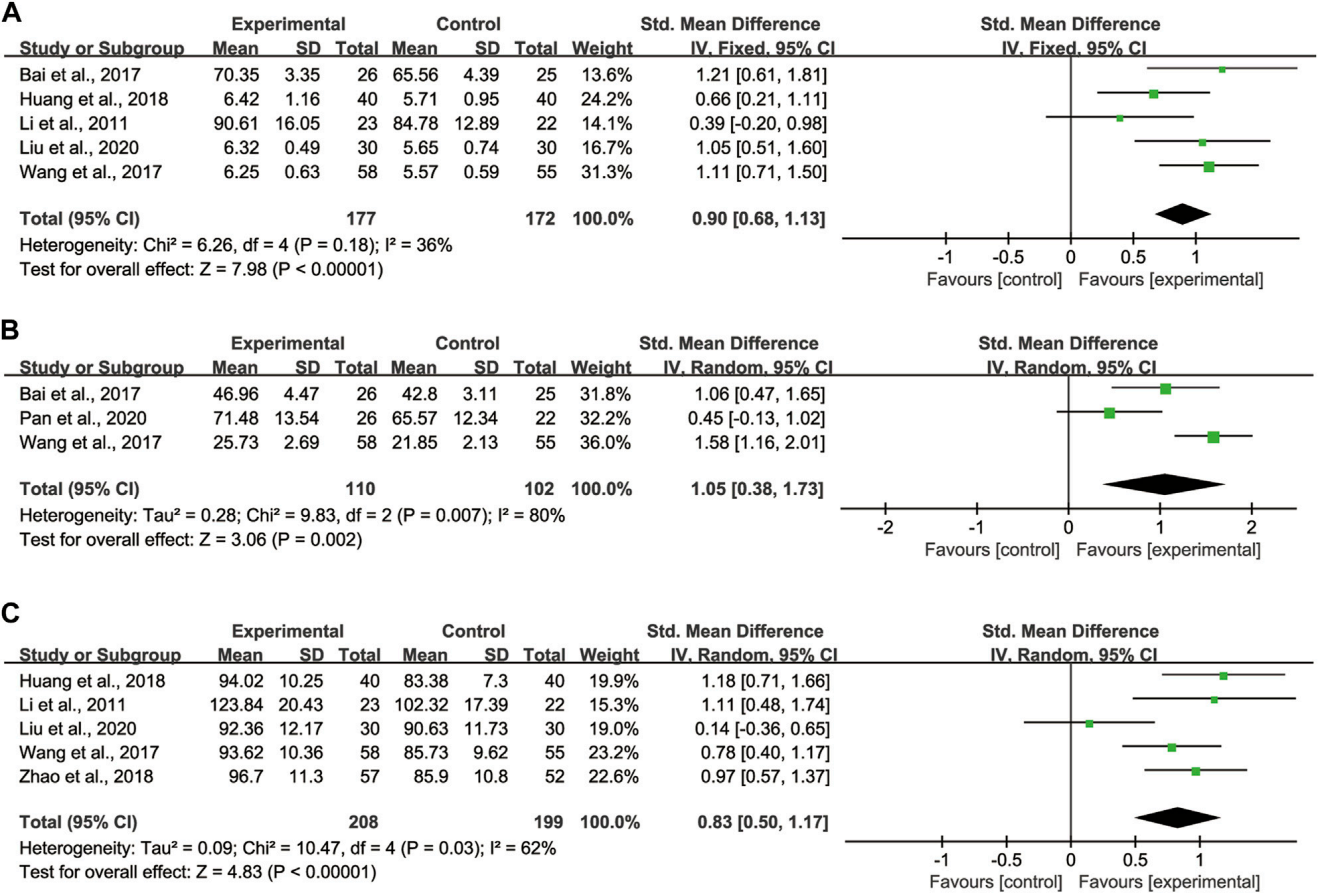
Forest plot of lung function: CHMs plus CTX treatment *versus* CTX treatment. **(A)** TLC, **(B)** DLCO, **(C)** MVV.

Regarding TLC, 5 studies (Li et al., 2011; Bai et al., 2017; Wang et al., 2017; Huang et al., 2018; Liu et al., 2020) with totaling 349 patients (177 in the experimental group, 172 in the control group) were reported, as these studies showed no significant heterogeneity (*I* ^*2*^ = 36%), we adopted the fixed effect model (Figure 5A). Pooled analysis showed that CHMs combined with CTX treatment had advantages in improving TLC compared with CTX treatment [SMD = 0.90, 95% CI: (0.68, 1.13), *p* < 0.00001].

Regarding DLCO, 3 studies (Bai et al., 2017; Wang et al., 2017; Pan et al., 2020) with totaling 212 patients (110 in the experimental group, 102 in the control group) were reported, as data heterogeneity test was high (*I* ^*2*^ = 80%), we adopted the random effect model (Figure 5B). Pooled analysis showed that CHMs combined with CTX treatment could significantly improve the DLCO of patients [SMD = 1.05, 95% CI: (0.38, 1.73), *p* = 0.002].

Regarding MVV, 5 studies (Li et al., 2011; Wang et al., 2017; Huang et al., 2018; Zhao et al., 2018; Liu et al., 2020) with totaling 407 patients (208 in the experimental group, 199 in the control group) were reported, as we noted significant heterogeneity in these studies (*I* ^*2*^ = 62%), the random effect model was adopted (Figure 5C). According to the pooled analysis results in MVV, we concluded that the CHMs combined with CTX group could improve the MVV of patients [SMD = 0.83, 95% CI: (0.50, 1.17), *p* < 0.00001].

##### Lungs HRCT integral evaluation

**FIGURE 6:**
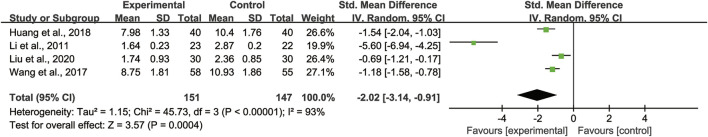
Forest plot of HRCT integral of lungs: CHMs plus CTX treatment *versus* CTX treatment.

4 studies (Li et al., 2011; Wang et al., 2017; Huang et al., 2018; Liu et al., 2020) with totaling 298 patients (151 in the experimental group, 147 in the control group) reported HRCT integral of lungs. We adopted the random effect model due to large heterogeneity (*I* ^*2*^ = 93%) (Figure 6). Pooled analysis showed that between CHMs combination group and control group existed a statistically difference, signifying that CHMs combined with CTX treatment could significantly reduce the HRCT integral of lungs [SMD = −2.02, 95% CI: (−3.14, −0.91), *p* = 0.0004].

#### Secondary outcomes

##### Clinical efficacy rate

**FIGURE 7:**
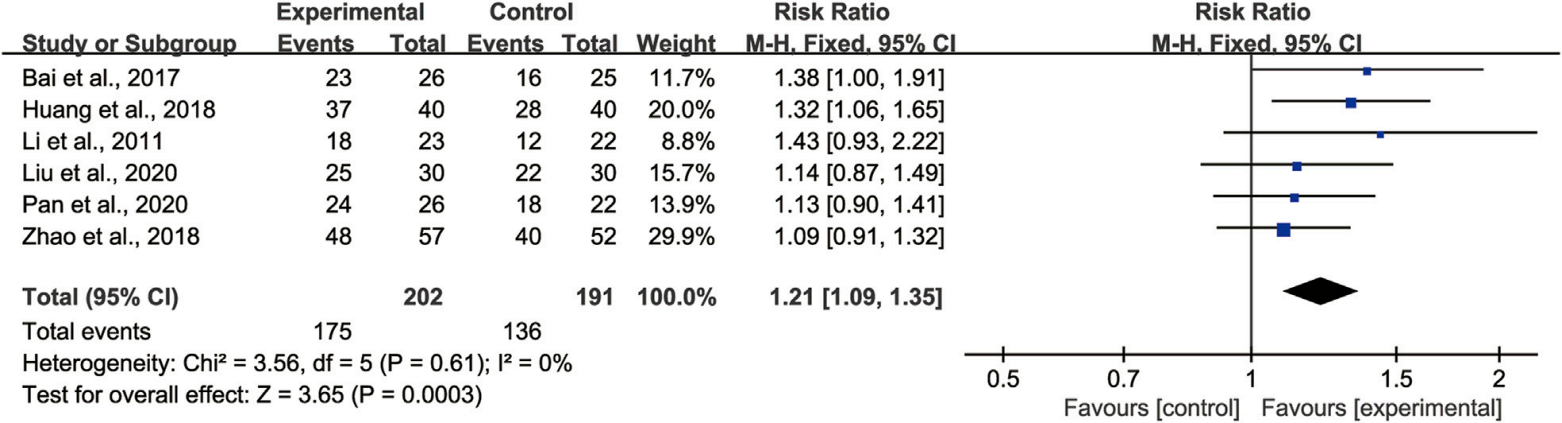
Forest plot of clinical efficacy rate: CHMs plus CTX treatment *versus* CTX treatment.

6 studies (Li et al., 2011; Bai et al., 2017; Huang et al., 2018; Zhao et al., 2018; Liu et al., 2020; Pan et al., 2020) with totaling 393 patients (202 in the experimental group, 191 in the control group) reported Clinical efficacy rates. The clinical efficacy rates referred to the proportion of clinical cure, markedly effective and improved patients in the total number after treatment. Clinical cure was defined as an 95% recovery of related clinical symptoms, markedly effective was defined as an 70%–94% recovery, and improved was 30%–69%, while invalid was <30%. We adopted the fixed effect model due to heterogeneity was not found (*I* ^*2*^ = 0%) (Figure 7). Pooled analysis showed that the CHMs combined with CTX group clinical efficacy rates were higher than the CTX group [RR = 1.21, 95% CI: (1.09, 1.35), *p* = 0.0003].

##### Inflammatory indicators

**FIGURE 8:**
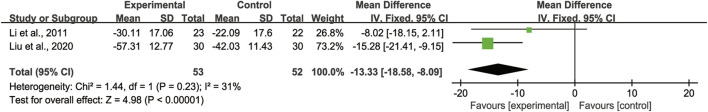
Forest plot of ESR: CHMs plus CTX treatment *versus* CTX treatment.

2 studies (Li et al., 2011; Liu et al., 2020) with totaling 105 patients (53 in the experimental group, 52 in the control group) reported improvement of ESR. We adopted the fixed effect model due to heterogeneity was not found (*I* ^*2*^ = 31%) (Figure 8). Pooled analysis indicated that CHMs combined with CTX treatment could effectively reduce the level of ESR compared with CTX treatment [WMD = −13.33, 95% CI: (−18.58, −8.09), *p* < 0.00001].

### Adverse event reporting

4 studies (Li et al., 2011; Bai et al., 2017; Zhao et al., 2018; Liu et al., 2020) provided AEs, among them, only 1 study (Zhao et al., 2018) mentioned that there were no AEs in both groups, the remaining 3 studies with totaling 156 patients (79 in the experimental group, 77 in the control group) reported AEs. Incidence of AEs occurred in 4/79 patients who received CHMs plus CTX treatment and 22/77 patients who received CTX treatment alone. Pooled analysis showed that there was no statistically significant difference in the incidence of AEs between the CHMs combination group and the control group (Supplementary Figure S1). In the CHMs combination group, the main AEs were stated as gastrointestinal discomfort (*n* = 3), leukopenia (*n* = 1). In the control group, the main AEs were stated as gastrointestinal discomfort (*n* = 3), liver dysfunction (*n* = 6), leukopenia (*n* = 6), cold symptoms (*n* = 4), dizziness and fatigue (*n* = 3). These AEs were transient and some improved without special treatment, some improved with symptomatic treatment. Because CTX treatment has obvious gastrointestinal reactions, liver dysfunction and leukopenia, the above AEs may be related to this reason. Thus, CHMs combined with CTX seems to be safe and does not increase AEs compared with CTX alone.

### Subgroup analysis

#### Subgroup analysis of lung function

**FIGURE 9:**
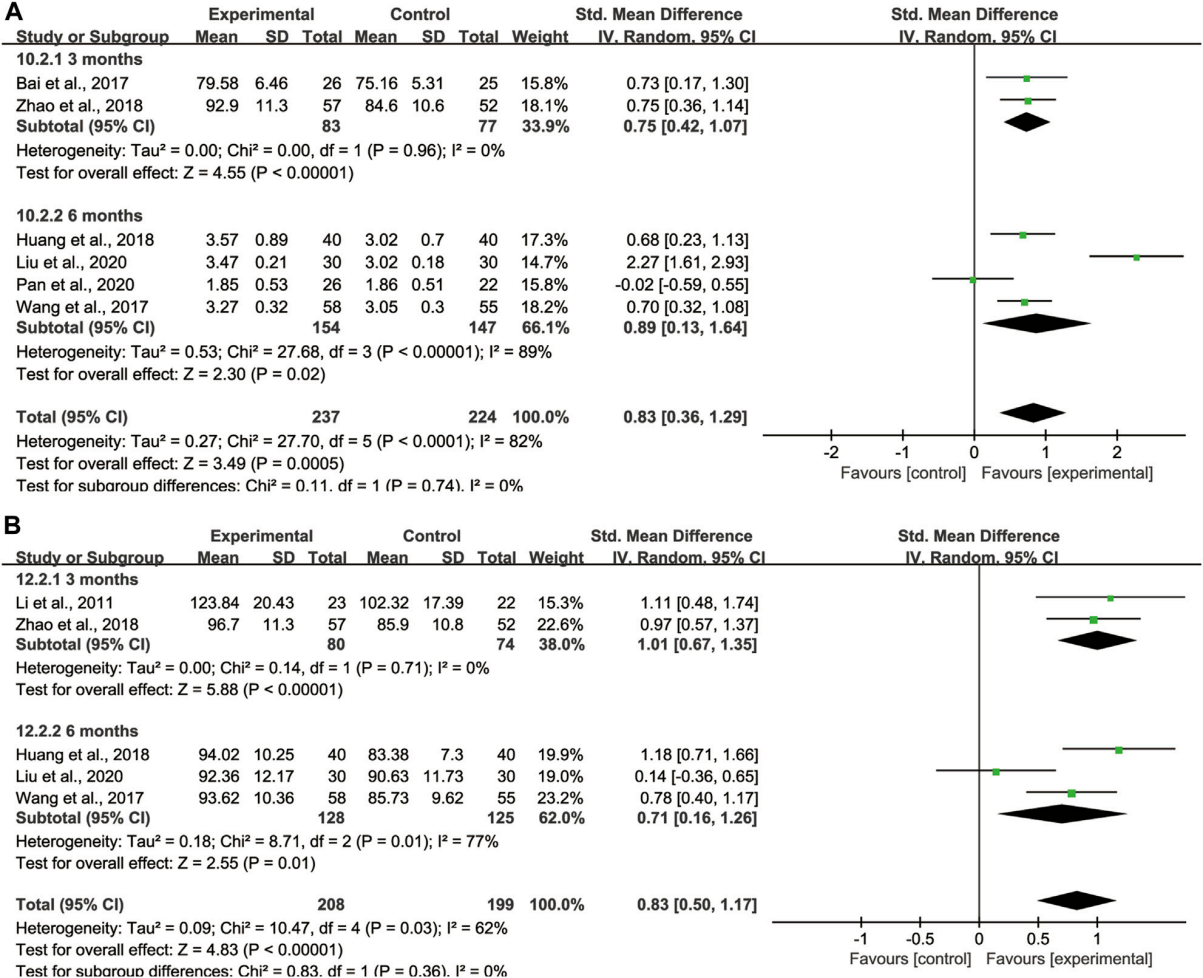
Subgroup analysis of lung function based on different treatment duration. **(A)** FVC, **(B)** MVV.

**FIGURE 10:**
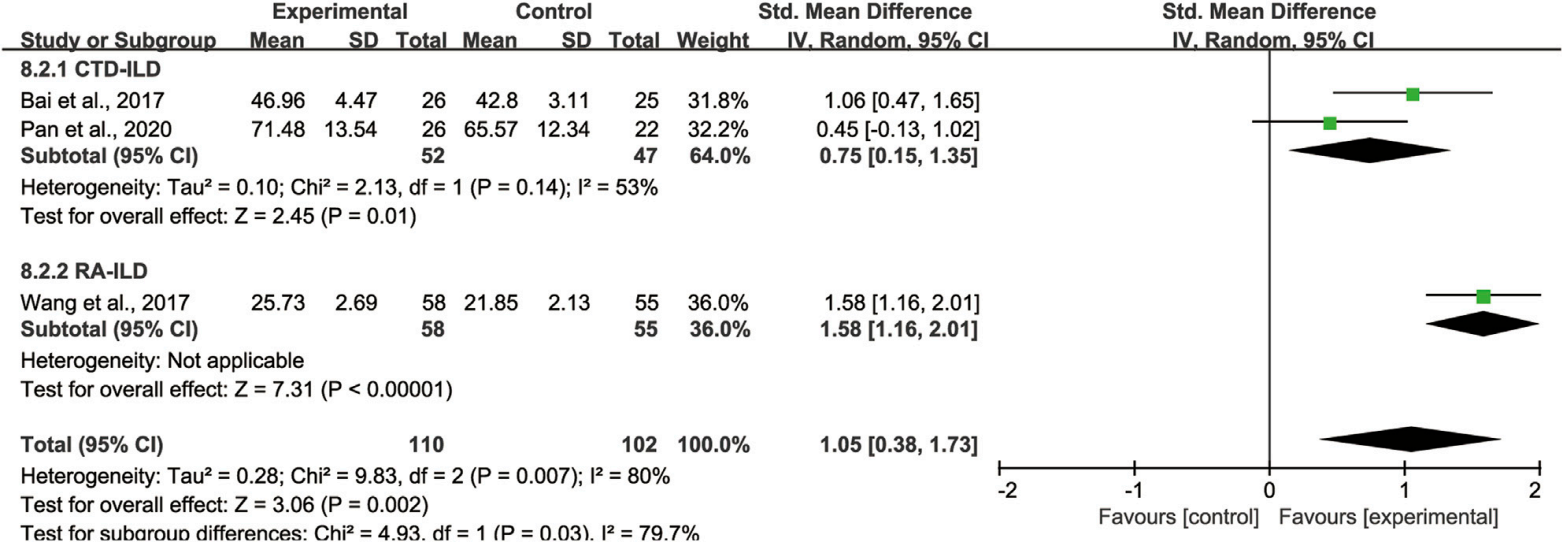
Subgroup analysis of DLCO based on different etiologies.

With regard to FVC, DLCO, and MVV, we also took subgroup analyses due to high heterogeneity to further evaluate whether the different treatment duration and primary disease could lead to the heterogeneity. For FVC and MVV, we divided them into 3 months treatment group and 6 months treatment group (Figures 9A, B). For DLCO, we grouped them according to different etiologies (Figure 10). Subgroup analysis eliminated the heterogeneity of FVC, DLCO and MVV, which may account for the partial heterogeneity. However, for FVC, different etiologies were not the sources of heterogeneity (Supplementary Figure S2), for MVV, it might be the sources of heterogeneity (Supplementary Figure S3).

#### Subgroup analysis of HRCT integral of lungs

**FIGURE 11:**
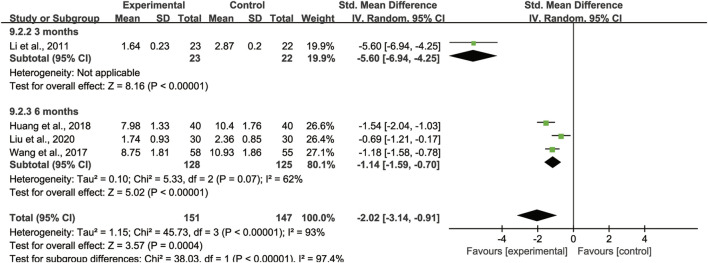
Subgroup analysis of HRCT integral of lungs based on different treatment duration.

Since there was large heterogeneity among the 4 studies of HRCT integral of lungs, we conducted a subgroup analysis to investigate the heterogeneity sources. We divided them into 3 months treatment group and 6 months treatment group according to the length of treatment course. Heterogeneity decreased after subgroup analyses with different treatment duration, which may account for the partial heterogeneity (Figure 11).

### Sensitivity analysis

**FIGURE 12:**
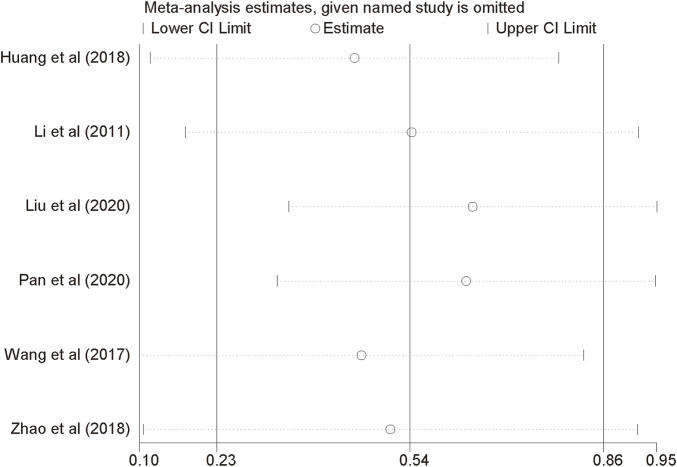
Sensitivity analysis plots of FEV1.

Since the reliability of the FEV1 result were affected by the huge heterogeneity, we took sensitivity analyses to investigate the heterogeneity sources. After eliminated the included trials one by one, it did not alter the overall results, which indicated that our results were stable and constant (Figure 12).

### Publication bias

We planned to use the clinical efficacy rate as the outcome index for Funnel plots analysis to examine the publication bias, but only 6 records met the requirements. Therefore, publication bias analysis was not performed.

### Description of the CHMs

A total of 5 herbal decoctions, 1 granule and 1 oral liquid were used in seven included studies. The herbal components number in the formulae varied from 4 to 15. To determine the characteristics of the CHMs botanical drugs included in the study, we summarized the specific drug ingredients and dosage in each original study (Table 2). Meanwhile, we ranked the frequency of botanical drugs use in the included studies (frequency ≥3) (Supplementary Table S3). In the included studies, the most commonly used botanical drugs were *Astragalus mongholicus* Bunge (Huangqi), *Angelica sinensis* (Oliv.) Diels (Danggui), *Glycyrrhiza uralensis* Fisch. ex DC. (Gancao), *Salvia miltiorrhiza* Bunge (Danshen), *Adenophora triphylla* (Thunb.) A.DC. (Nanshashen), and *Schisandra chinensis* (Turcz.) Baill. (Wuweizi).

**TABLE 2:** Components of Chinese herbal medicine used in the included studies.

Study Id	Prescription name	Source	Compositions	Usage of preparations	Preparations	Quality control reported?	Chemical analysis reported?
Bai et al. (2017)	Removing both Phlegm and Blood Stasis Formula	Chinese PLA General Hospital	*Fritillaria thunbergii* Miq. [Liliaceae; Fritillariae bulbus] 15g; *Curcuma aeruginosa* Roxb. [Zingiberaceae; Curcumae aeruginosae rhizoma] 10g; *Coix lacryma-jobi var. ma-yuen *(Rom.Caill.) Stapf [Poaceae; Coicis semen] 30g; *Poria cocos* (Schw.) Wolf [Polyporaceae; Poria] 15g; *Panax notoginseng* (Burkill) F.H.Chen [Araliaceae; Notoginseng radix et rhizoma] 3g; *Trichosanthes kirilowii *Maxim*.* [Cucurbitaceae; Trichosanthis radix] 15g; *Glycyrrhiza uralensis* Fisch. ex DC. [Fabaceae; Glycyrrhizae radix et rhizoma] 6 g	200 ml bid po	Decoction	N	N
Huang et al. (2018)	Yangyin Tongbi Formula	Guangzhou University of TCM	*Codonopsis pilosula* (Franch.) Nannf. [Campanulaceae; Codonopsis radix] 30g; *Astragalus mongholicus* Bunge [Fabaceae; Astragali radix] 30g; *Rehmannia glutinosa* (Gaertn.) DC. [Orobanchaceae; Rehmanniae radix praeparata] 30g; *Schisandra chinensis* (Turcz.) Baill. [Schisandraceae; Schisandrae chinensis fructus] 20g; *Aster tataricus* L.f. [Asteraceae; Asteris radix et rhizoma] 20g; *Angelica sinensis* (Oliv.) Diels [Apiaceae; Angelicae sinensis radix] 20g; *Eclipta prostrata* (L.) L. [Asteraceae; Ecliptae herba] 20g; *Adenophora triphylla* (Thunb.) A.DC. [Campanulaceae; Adenophorae radix] 15g; *Salvia miltiorrhiza* Bunge [Lamiaceae; Salviae miltiorrhizae radix et rhizoma] 15 g	200 ml bid po	Decoction	N	N
Li et al. (2011)	Fangxian Decoction	The First Affiliated Hospital of Henan College of TCM	*Astragalus mongholicus* Bunge [Fabaceae; Astragali radix] 20g; *Atractylodes macrocephala* Koidz. [Asteraceae; Atractylodis macrocephalae rhizoma] 12g; *Adenophora triphylla* (Thunb.) A.DC. [Campanulaceae; Adenophorae radix] 15g; *Ophiopogon japonicus* (Thunb.) Ker Gawl. [Asparagaceae; Ophiopogonis radix] 15g; *Schisandra chinensis* (Turcz.) Baill. [Schisandraceae; Schisandrae chinensis fructus] 10g; *Rehmannia glutinosa* (Gaertn.) DC. [Orobanchaceae; Rehmanniae radix] 10g, *Angelica sinensis* (Oliv.) Diels [Apiaceae; Angelicae sinensis radix] 10g; *Spatholobus suberectus* Dunn [Fabaceae; Spatholobi caulis] 30g; *Salvia miltiorrhiza* Bunge [Lamiaceae; Salviae miltiorrhizae radix et rhizoma] 30g; *Fritillaria cirrhosa* D.Don [Liliaceae; Fritillariae cirrhosae bulbus] 15g; *Morus alba* L. [Moraceae; Mori cortex] 15g; *Pheretima aspergillum* (E. Perrier) [Megascolecidae; Pheretima] 15g; *Bombyx mori* Linnaeus [Silkworm pilgrimaging; Bombyx batryticatus] 15g; *Centella asiatica* L.) Urb. [Apiaceae; *Centella asiatica*] 30g; *Tripterygium wilfordii* Hook.f. [Celastraceae; Tripterygium wilfordii radix] 10 g	200 ml bid po	Decoction	N	N
Liu et al. (2020)	Tongbi Granules	The First Affiliated Hospital of Hunan University of TCM	*Astragalus mongholicus* Bunge [Fabaceae; Astragali radix]; *Angelica sinensis* (Oliv.) Diels [Apiaceae; Angelicae sinensis radix]; *Paeonia lactiflora* Pall. [Paeoniaceae; Paeoniae radix alba]; *Ligusticum chuanxiong* Hort. [Apiaceae; Chuanxiong rhizoma]; *Bombyx mori* Linnaeus [Silkworm pilgrimaging; Bombyx batryticatus]; *Buthus martensi* Karsch [Buthidae; Scorpion]; *Boswellia sacra* Flück. [Burseraceae; Frankincense]; *Commiphora myrrha* (T.Nees) Engl. [Burseraceae; Myrrh]; *Neolitsea cassia* L.) Kosterm. [Lauraceae; Cinnamomi ramulus]; *Phellodendron amurense* Rupr. [Rutaceae; Phellodendri cortex]; *Glycyrrhiza uralensis* Fisch. ex DC. [Fabaceae; Glycyrrhizae radix et rhizoma]	6 g bid po	Granule	N	N
Pan et al. (2020)	Shengbu Zongqi Formula	Dongying Shengli Hospital	*Codonopsis pilosula* (Franch.) Nannf. [Campanulaceae; Codonopsis radix] 30g; *Astragalus mongholicus* Bunge [Fabaceae; Astragali radix] 30g; *Actaea cimicifuga* L. [Ranunculaceae; Cimicifugae rhizoma] 10g; *Bupleurum chinense* DC. [Apiaceae; Bupleuri radix] 10g; *Anemarrhena asphodeloides* Bunge [Asparagaceae; Anemarrhenae rhizoma] 10g; *Platycodon grandiflorus* (Jacq.) A.DC. [Campanulaceae; Platycodonis radix] 10g; *Angelica sinensis* (Oliv.) Diels [Apiaceae; Angelicae sinensis radix] 15g; *Ligusticum chuanxiong* Hort. [Apiaceae; Chuanxiong rhizoma] 15g; *Pueraria montana var. lobata* (Willd.) Maesen & S.M.Almeida ex Sanjappa & Predeep [Fabaceae; Pueraria radix] 15 g	200 ml bid po	Decoction	N	N
Wang et al. (2017)	Yiqi Yangyin Tongbi Formula	Henan Provincial Hospital of TCM	*Panax ginseng* C.A.Mey. [Araliaceae; Ginseng radix et rhizoma] 10g; *Astragalus mongholicus* Bunge [Fabaceae; Astragali radix] 30g; *Rehmannia glutinosa* (Gaertn.) DC. [Orobanchaceae; Rehmanniae radix praeparata] 30g; *Schisandra chinensis* (Turcz.) Baill. [Schisandraceae; Schisandrae chinensis fructus] 6g; *Aster tataricus* L.f. [Asteraceae; Asteris radix et rhizoma] 10g; *Morus alba* L. [Moraceae; Mori cortex] 12g; *Angelica sinensis* (Oliv.) Diels [Apiaceae; Angelicae sinensis radix] 10g; *Eclipta prostrata* L.) L. [Asteraceae; Ecliptae herba] 20g; *Adenophora triphylla* (Thunb.) A.DC. [Campanulaceae; Adenophorae radix] 20g; *Salvia miltiorrhiza* Bunge [Lamiaceae; Salviae miltiorrhizae radix et rhizoma] 20g; *Curcuma longa* L. [Zingiberaceae; Curcumae longae rhizoma] 12g; *Citrus maxima* (Burm.) Merr. [Rutaceae; Citri grandis exocarpium] 15g; *Glycyrrhiza uralensis* Fisch. ex DC. [Fabaceae; Glycyrrhizae radix et rhizoma] 6 g	200 ml bid po	Decoction	N	N
Zhao et al. (2018)	Sangzhu Ziyin Oral Liquid	Yueyang Hospital of Integrated Traditional Chinese and Western Medicine	*Morus alba* L. [Moraceae; Mori fructus]; *Angelica dahurica* (Hoffm.) Benth. & Hook.f. ex Franch. & Sav. [Apiaceae; Angelica dahuricae radix]; *Dioscorea oppositifolia* L. [Dioscoreaceae; Dioscoreae rhizoma]; *Pteria martensii* (Dunker) [Pteriidae; Margarita]	10 ml bid po	Oral Liquid	N	N

PLA, Chinese People’s Liberation Army; TCM, traditional chinese medicine; N, NO.

## Discussion

CTD-ILD is one of the serious complications of CTD patients, which has been a challenging disorder. At present, the unified and standardized diagnosis and treatment guidelines have not been established internationally, and there are no officially approved drugs for CTD-ILD treatment. The treatment of CTD-ILD is mainly aimed at the treatment of the primary disease and symptomatic treatment of respiratory symptoms. Many disease-modifying antirheumatic drugs (DMARDs) for the CTD primary disease treatment have adverse reactions to varying degrees, such as increasing the risk of pulmonary infection, pulmonary sarcoidosis and interstitial pneumonia. Meanwhile, the efficacy of DMARDs for CTD-ILD clinical application is very limited, especially for patients who have severe lung damage (Brummaier et al., 2013). Although for idiopathic interstitial lung disease, the use of antifibrotic targeted drugs such as nidanib and pirifenidone have achieved certain clinical results. However, evidence-based medicine is still lacking. Therefore, we urgently need standardized therapeutic measures or new treatment methods.

Nowadays, CHMs is widely used in the treatment of CTD-ILD to improve its symptoms and signs, and to control disease progression. According to the TCM theory, the pathogenesis of CTD-ILD is related to deficiency of both lung and kidney, and blood stasis obstructing collaterals, which can be divided into deficiency syndrome, excess syndrome, deficiency and excess mixed syndrome. Deficiency of lung and kidney can lead to cough and shortness of breath, while blood stasis can lead to aggravation of the disease. The two often interact as cause and effect and promote each other. Therefore, it is often treated with TCM for nourishing lung and kidney, promoting blood circulation and removing blood stasis, and usually achieves good results (Chen, 2018; Qu et al., 2021). This study had made statistics on the commonly used TCM for the treatment of CTD-ILD, among which *A. mongholicus* Bunge (Huangqi) and *G. uralensis* Fisch. ex DC. (Gancao) replenish Qi, *A. sinensis* (Oliv.) Diels (Danggui) replenish Blood, *A. triphylla* (Thunb.) A.DC. (Nanshashen) nourish Yin and *S. miltiorrhiza* Bunge (Danshen) promote blood circulation. In recent years, more and more RCTs have used CHMs to treat CTD-ILD, which provides an opportunity for comprehensive and objective evaluation of TCM treatment methods.

### Summary of the evidence

This study reviewed seven RCTs involving 506 patients. Our meta-analysis found the following: 1) CHMs combined with CTX could improve the clinical efficacy rate compared with the CTX alone; 2) CHMs combined with CTX could improve lung function compared with CTX, including VC, FVC, FEV1, TLC, DLCO and MVV; 3) CHMs combined with CTX was superior to pure CTX in improving HRCT integral; 4) CHMs combined with CTX was superior to CTX alone in improving ESR; 5) CHMs combined with CTX did not increase AEs compared with pure CTX. These results suggest that TCM can be considered a potentially valid and safe drug in the management of CTD-ILD patients.

At present, many basic studies have also confirmed that CHMs can treat CTD-ILD through multiple targets and multiple pathways. It is believed that CHMs can selectively regulate JAK2/STATs signaling pathway, restore helper T cells 17 (Th17)/regulatory T cell (Treg) balance to reduce inflammation and fibrosis in pulmonary interstitial lesions in SLE mice (Tan et al., 2022). Studies have confirmed that CHMs can act on Notch signaling pathway to effectively reduce the degree of arthritis in RA-ILD rats and improve the inflammatory infiltration of lung tissue (Li H. et al., 2021). At the same time, some scholars believe that CHMs can significantly inhibit the high expression of IL-6, IL-17, KL-6, and effectively delay the progression of RA-ILD (Xi et al., 2022). Therefore, CHMs can theoretically alleviate respiratory symptoms, reduce inflammation, and improve lung function, with a significant therapeutic effect on CTD-ILD.

### Limitations

Several limitations in the meta-analysis should also be considered. First, although we had searched both Chinese and English databases, all the included trials in this study were conducted in China, which may present selection bias and limit the results wide application. Second, none of the included trials in this study provided allocation or blinding information, so the methodological quality of literature was found to be generally low, which may affect the reliability and credibility of its findings, make it prone to draw false negative or false positive conclusions. Third, most trials did not calculate the formal pretrial sample size. The trials with small sample sizes appear to be one risk in exaggerating intervention benefits. Fourth, heterogeneity among the studies should be considered seriously when interpreting the results. Different treatment duration, different disease duration, different ingredients and functions of TCM, and so on, may produce a certain heterogeneity, thus reducing the robustness of the conclusion and influencing the results. Finally, owing to being highly variable in composition and dosage of CHMs, and different measures of outcome indicators, it is difficult to assess the efficacy of a specific CHM by performing pooling analysis. However, our study showed that CHMs demonstrates good potential in treating CTD-ILD. Therefore, further research is needed to solve this limitation and CHMs treat CTD-ILD possible mechanisms need to be explored in the future.

### Implications for research

Based on the above limitations, some recommendations are suggested for further studies. First, blinding, allocation concealment, and random sequence generation should all be strictly implemented in future studies. We recommend that RCTs investigating CHM (Flower et al., 2012), CONSORT 2010 statement (Schulz et al., 2010), and CONSORT Extension for CHM Formulas 2017 (Cheng et al., 2017) should be used as the guidelines when the designing, registering, and reporting of further RCTs of CTD-ILD. Second, AEs were not reported in many RCTs. Therefore, whether the AEs exists should be reported in future studies based on the adverse reactions standard format established by Bian et al. (2010). Longer studies and clinical trials should be conducted to confirm the long-term safety of CHM for CTD-ILD. Third, we advise to establish a curative effect evaluation system conforming to the characteristics of CHM and explore practical and sensitive indicators of CHM.

### Implications for practice

Over the past few decades, the use of CHMs in treating CTD-ILD has increased. However, the choice of CHMs is empirical and there is a lack of consensus among clinicians. The evidence available from our study demonstrated the effectiveness and safety of CHM therapy for CTD-ILD. The most commonly used botanical drugs such as *A. mongholicus* Bunge (Huangqi), *A. sinensis* (Oliv.) Diels (Danggui), *G. uralensis* Fisch. ex DC. (Gancao), *S. miltiorrhiza* Bunge (Danshen), *A. triphylla* (Thunb.) A.DC. (Nanshashen), and *S. chinensis* (Turcz.) Baill. (Wuweizi) should be considered further in the development of Chinese herbal prescription for CTD-ILD. Thus, following the principle of high-frequency medication in the treatment of CTD-ILD by CHMs, their treatment principles can guide to CHM treatment for CTD-ILD and improve the clinical efficacy and safety, and can help implement individualized herbal prescriptions in future clinical practice.

## Conclusion

In general, the evidence in this study supports the fact that CHMs combined with CTX may be a more effective strategy on the treatment of CTD-ILD in the clinic. For CTD-ILD patients, compared with the CTX alone, CHMs combined with CTX can increase the clinical efficacy rates, improve lung function and HRCT integral, reduce the level of ESR, with no increased AEs incidence. However, because the quality of the included literature is relatively poor, our conclusions should be interpreted with some caution. Therefore, we also need large-scale and more multi-center RCTs to objective and comprehensive evaluation of the effectiveness and safety of CHMs in patients with CTD-ILD in future.

## Data Availability

The original contributions presented in the study are included in the article/Supplementary Material, further inquiries can be directed to the corresponding author.
