# Supplementary material for: Efficacy and safety of Chinese herbal medicines combined with cyclophosphamide for connective tissue disease-associated interstitial lung disease: A meta-analysis of randomized controlled trials
Source: Front Pharmacol. 2023 Feb 23;14:1064578. doi: 10.3389/fphar.2023.1064578 (PMC9995361; doi:10.3389/fphar.2023.1064578)
Supplement: Supplementary file 1 [file Table3.DOC]

Embase

Session Results

.......................................................

No. Query Results Results Date

#36. #13 AND #21 AND #30 AND #35 28 Aug 2022

#35. #31 OR #32 OR #33 OR #34 1,324,066 28 Aug 2022

#34. 'random':ti,ab 396,180 28 Aug 2022

#33. 'double-blind':ti,ab 215,315 28 Aug 2022

#32. 'placebo':ti,ab 346,381 28 Aug 2022

#31. 'randomized controlled trial'/exp 727,452 28 Aug 2022

#30. #22 OR #23 OR #24 OR #25 OR #26 OR #27 OR #28 OR 78,916 28 Aug 2022

#29

#29. 'chinese and western medicine':ti,ab 1,312 28 Aug 2022

#28. 'traditional chinese medicine':ti,ab 34,573 28 Aug 2022

#27. 'medicine, chinese traditional':ti,ab 11 28 Aug 2022

#26. 'chinese plant extracts':ti,ab 3 28 Aug 2022

#25. 'chinese herbal drugs':ti,ab 224 28 Aug 2022

#24. 'chinese traditional medicine':ti,ab 1,505 28 Aug 2022

#23. 'drugs, chinese herbal':ti,ab 9 28 Aug 2022

#22. 'chinese medicine'/exp 65,229 28 Aug 2022

#21. #14 OR #15 OR #16 OR #17 OR #18 OR #19 OR #20 105,240 28 Aug 2022

#20. 'lung diseases, interstitial':ti,ab 14 28 Aug 2022

#19. 'interstitial pneumonias':ti,ab 1,277 28 Aug 2022

#18. 'interstitial pneumonia':ti,ab 12,824 28 Aug 2022

#17. 'pneumonia, interstitial':ti,ab 105 28 Aug 2022

#16. 'lung disease, interstitial':ti,ab 40 28 Aug 2022

#15. 'interstitial lung diseases':ti,ab 4,469 28 Aug 2022

#14. 'interstitial lung disease'/exp 102,214 28 Aug 2022

#13. #1 OR #2 OR #3 OR #4 OR #5 OR #6 OR #7 OR #8 OR 540,027 28 Aug 2022

#9 OR #10 OR #11 OR #12

#12. 'arthritis, rheumatoid':ti,ab 553 28 Aug 2022

#11. 'dermatopolymyositis':ti,ab 160 28 Aug 2022

#10. 'scleroderma, systemic':ti,ab 394 28 Aug 2022

#9. 'lupus erythematosus, systemic':ti,ab 334 28 Aug 2022

#8. 'sjogrens syndrome':ti,ab 375 28 Aug 2022

#7. 'connective tissue disease':ti,ab 12,638 28 Aug 2022

#6. 'dermatomyositis'/exp 19,299 28 Aug 2022

#5. 'systemic sclerosis'/exp 36,897 28 Aug 2022

#4. 'systemic lupus erythematosus'/exp 110,036 28 Aug 2022

#3. 'sjoegren syndrome'/exp 27,150 28 Aug 2022

#2. 'rheumatoid arthritis'/exp 241,404 28 Aug 2022

#1. 'connective tissue disease'/exp 538,451 28 Aug 2022

.......................................................
