# Supplementary material for: Efficacy and safety of Chinese herbal medicines combined with cyclophosphamide for connective tissue disease-associated interstitial lung disease: A meta-analysis of randomized controlled trials
Source: Front Pharmacol. 2023 Feb 23;14:1064578. doi: 10.3389/fphar.2023.1064578 (PMC9995361; doi:10.3389/fphar.2023.1064578)
Supplement: Supplementary file 2 [file Table1.DOC]

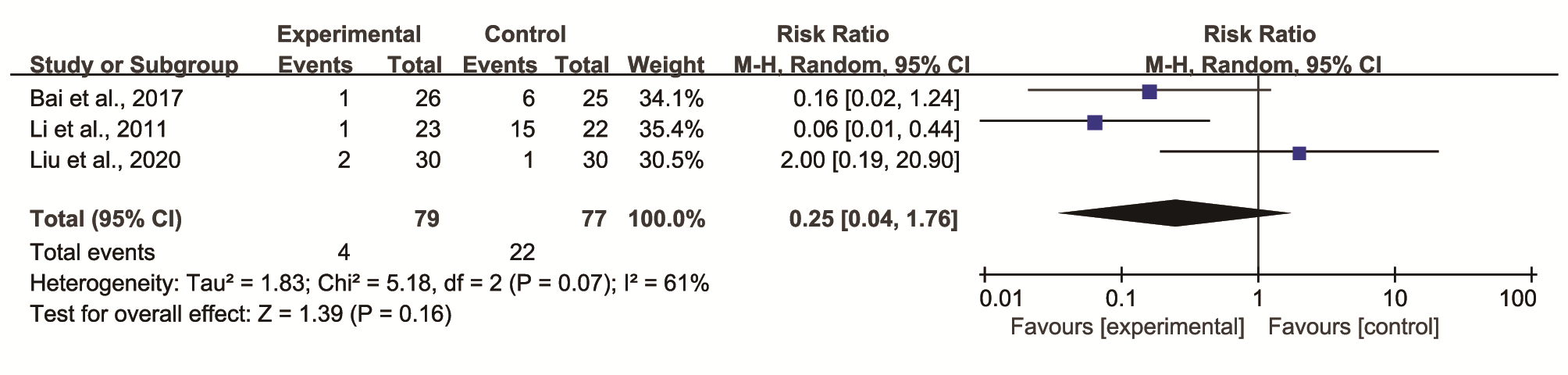


Supplementary Figure S1 | Forest plot of AEs.


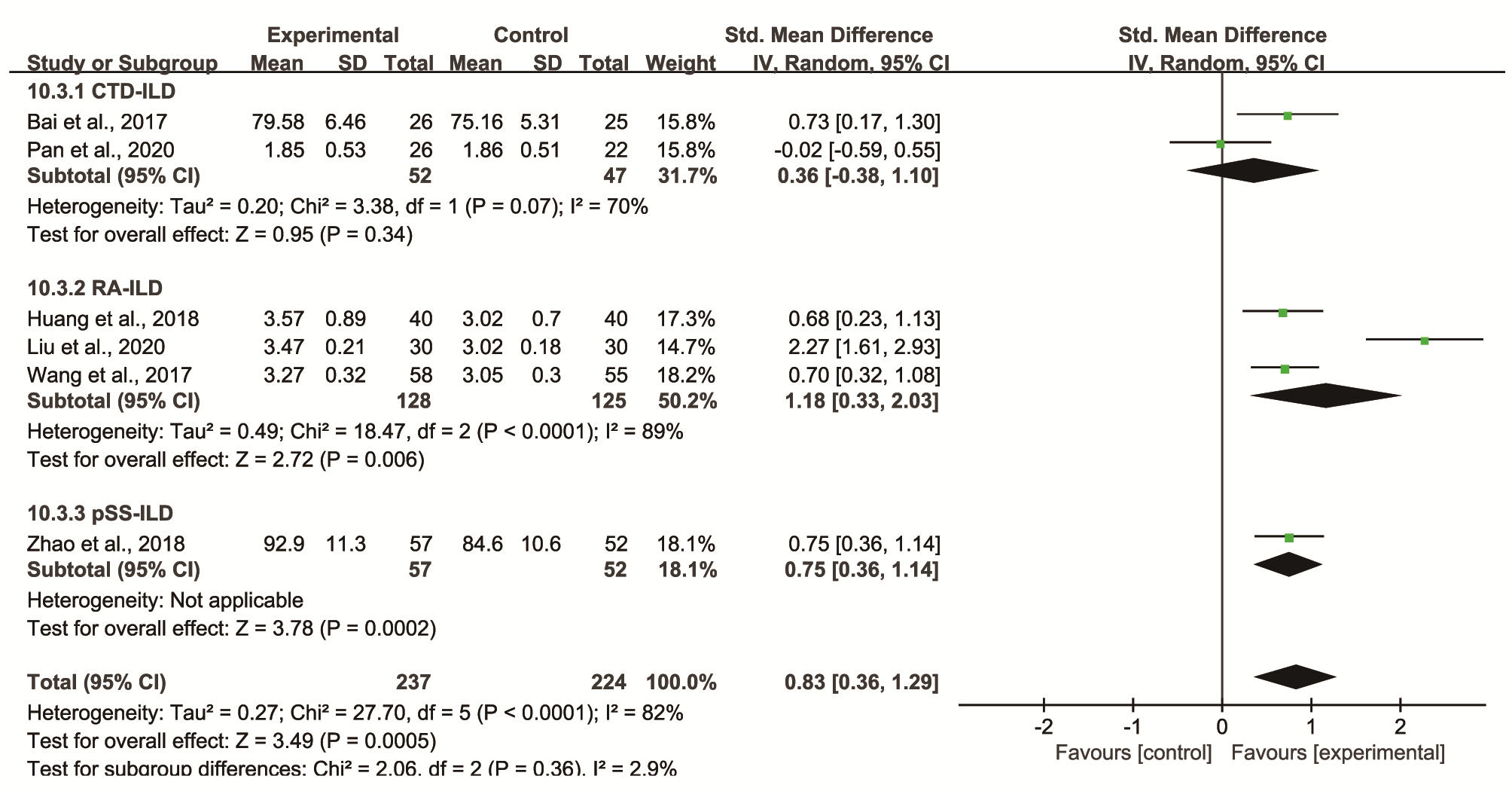


Supplementary Figure S2 | Subgroup analysis of FVC based on different etiologies.


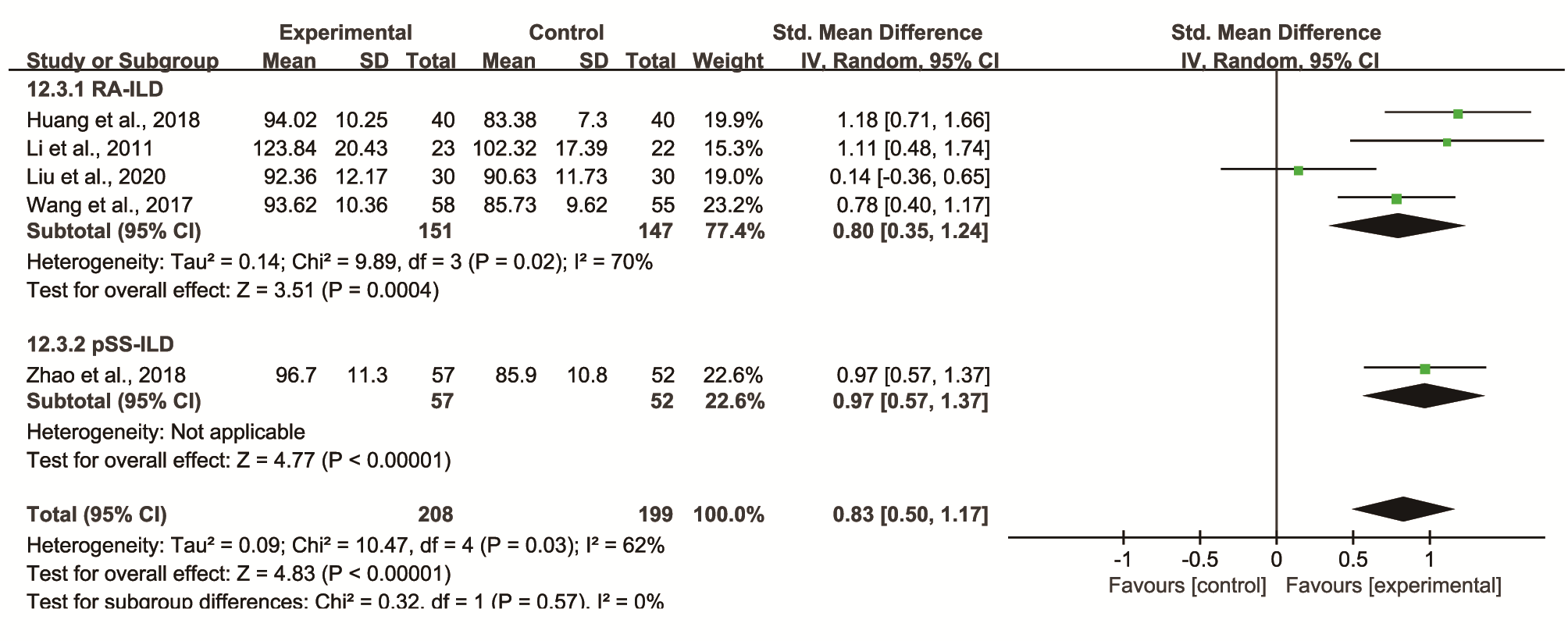


Supplementary Figure S3 | Subgroup analysis of MVV based on different etiologies.
