# Supplementary material for: Efficacy and safety of Chinese herbal medicines combined with cyclophosphamide for connective tissue disease-associated interstitial lung disease: A meta-analysis of randomized controlled trials
Source: Front Pharmacol. 2023 Feb 23;14:1064578. doi: 10.3389/fphar.2023.1064578 (PMC9995361; doi:10.3389/fphar.2023.1064578)
Supplement: Supplementary file 4 [file Table4.DOCX]

**SUPPLEMENTARY TABLE** **S3** Most commonly used ingredients in CHMs therapy.

| **Chinese name** | **Accepted scientific name** | **English name** | **Family** | **N/7(%)** |
| --- | --- | --- | --- | --- |
| Huangqi | *Astragalus mongholicus* Bunge | Milkvetch root | Fabaceae | 5(71%) |
| Danggui | *Angelica sinensis* (Oliv.) Diels | Chinese angelica | Apiaceae | 5(71%) |
| Gancao | *Glycyrrhiza uralensis* Fisch. ex DC. | Liquorice root | Fabaceae | 3(43%) |
| Danshen | *Salvia miltiorrhiza* Bunge | chinese salvia | Lamiaceae | 3(43%) |
| Nanshashen | *Adenophora triphylla* (Thunb.) A.DC. | ladybell root | Campanulaceae | 3(43%) |
| Wuweizi | *Schisandra chinensis* (Turcz.) Baill. | Chinese magnoliavine fruit | Schisandraceae | 3(43%) |
